# Active‐ion‐gated room temperature acetone gas sensing of ZnO nanowires array

**DOI:** 10.1002/EXP.20220065

**Published:** 2022-10-14

**Authors:** Junmeng Guo, Jiahui Gan, Haoran Ruan, Xiaobo Yuan, Chuiyun Kong, Yang Liu, Meiying Su, Yabing Liu, Wei Liu, Bao Zhang, Yongle Zhang, Gang Cheng, Zuliang Du

**Affiliations:** ^1^ Key Lab for Special Functional Materials Ministry of Education National & Local Joint Engineering Research Center for High‐Efficiency Display and Lighting Technology School of Materials Science and Engineering and Collaborative Innovation Center of Nano Functional Materials and Applications Henan University Kaifeng China

**Keywords:** active ions, gas sensors, room temperature, metal oxide semiconductor, ZnO

## Abstract

Reducing the high operation temperature of gas sensor to room temperature (RT) have attracted intense interests for its distinct preponderances, including energy‐saving and super stability, which presents great prospects in commercial application. The exciting strategies for RT gas sensing, such as unique materials with activated surface or light activation, do not directly modulate the active ions for gas sensing, limiting the RT gas sensing performances. Here, an active‐ion‐gated strategy has been proposed for RT gas sensing with high performance and low power consumption, in which gas ions in triboelectric plasma are introduced into metal oxide semiconductor (MOS) film to act as both floating gate and active sensing ions. The active‐ion‐gated ZnO nanowires (NWs) array shows a sensitivity of 38.3% to 10 ppm acetone gas at RT, and the maximum power consumption is only 4.5 mW. At the same time, the gas sensor exhibits excellent selectivity to acetone. More importantly, the response (recovery) time of this sensor is as low as 11 s (25 s). It is found that OH^−^(H_2_O)_4_ ions in plasma are the key for realizing RT gas sensing ability, and an accompanied resistive switch is also observed. It is considered that the electron transfer between OH^−^(H_2_O)_4_ and ZnO NWs will forms a hydroxyl‐like intermediate state (OH*) on the top of Zn^2+^, leading to the band bending of ZnO and activating the reactive O_2_^−^ ions on the oxygen vacancies. The active‐ion‐gated strategy proposed here present a novel exploration to achieving RT gas sensing performance of MOS by activating sensing properties at the scale of ions or atoms.

## Graphical abstract

This paper demonstrates a novel active‐ion‐gated strategy to active the room temperature (RT) gas sensing performance of ZnO NWs by self‐powered triboelectric plasma system in ambient environment. The activated ZnO NWs shows considerable sensitivity, excellent selectivity, and rapid response to acetone gas at RT with maximum power consumption only 4.5 mW.

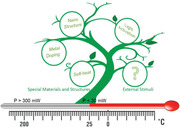

## 1 INTRODUCTION

Gas sensors can detect trace amounts of toxic and volatile organic gases (VOCs), which play an important role in industrial production, environmental monitoring, and public safety.^[^ ^1^, ^2^ ^]^ The chemo‐resistive gas sensor with metal oxide semiconductors (MOS) as the sensing layer demonstrates the advantages of high sensitivity, easy fabrication and operation, low cost, and miniaturization.^[^ ^3^ ^]^ As the earliest commercial gas sensor, the MOS gas sensors still occupy the largest share in the present market. However, traditional MOS gas sensors usually work at a high temperature (>200°C), which first cause the large power consumption.^[^ ^4^ ^]^ The power consumption of heating unit usually accounts for more than 90% of total power consumption of gas sensor. Furthermore, high temperature leads to the further growth of oxide particles and grain boundaries, which seriously affects the stability of gas sensing and reduces the lifetime of the sensor. It has been widely accepted that the adsorption/desorption of O_2_^−^ at defects is governing the sensing mechanism for MOS gas sensor. Taking the detection of reductive acetone gas as an example,^[^ ^5^ ^]^ O_2_ ionizes into O_2_^−^ by capturing an electron (O_2_ + e → O_2_^−^) from the oxygen vacancy (V_o_) on the surface of ZnO, and then the O_2_^−^ species reacts with acetone to realize the detection of acetone. The higher temperature is, the more active ions adsorbed would be produced, and more possible the redox reaction would take place. Hence, how to increase the numbers and reaction rate of adsorbed O_2_^−^ at room temperature (RT) is the main issue that needs to be solved in developing energy‐saving gas sensors.

**FIGURE 1:**
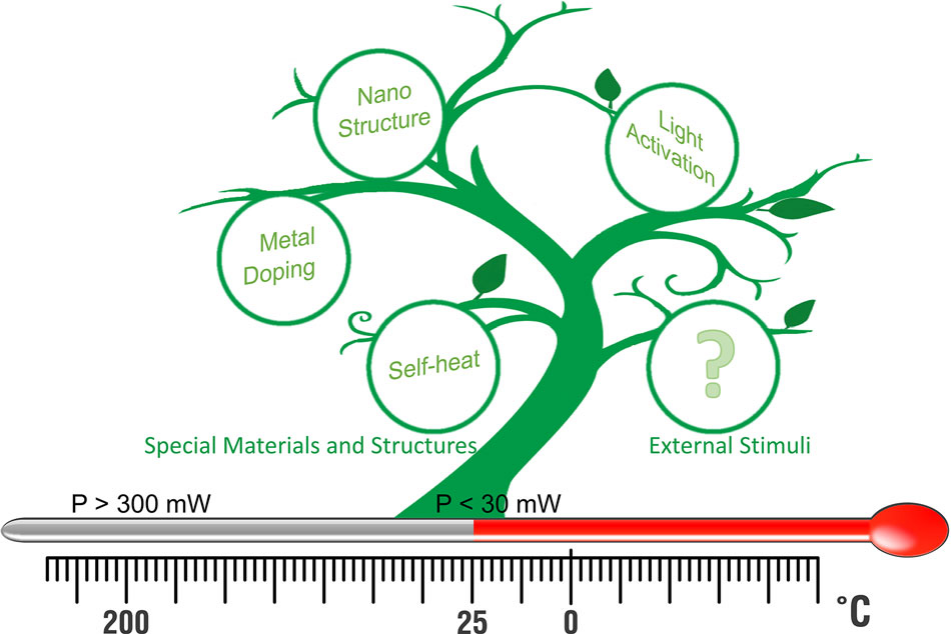
Research progress of metal oxide room temperature gas sensors

Until now, the reported RT sensing strategies are mainly divided into two types: one is that the sensor itself has RT sensing capability by designing unique materials and structures, and the other is that the sensor endows with the RT sensing capability by external stimuli (Figure 1). The first one mainly includes unique nanostructures, metal doping, and self‐heating effects.^[^ ^6^, ^7^, ^8^ ^]^ Some nanomaterials have high specific surface areas and more active sites, which imply a small change of adsorbed O_2_^−^ can significantly alter their electrical properties. For example, the conductance is controlled by the potential barrier height in ultralow dimensional nanomaterials, which shows an exponentially high sensitivity to the adsorbed O_2_^−^ species. When one or more dimensions of nanomaterials is less than twice Debye length (the width of the depletion layer), the ZnO or SnO_2_ can realize RT sensing to CO, H_2_S, ethanol, and acetone due to their ultrasmall size nature. ^[^ ^9^, ^10^ ^]^ Since the gas sensing process only requires a few O_2_^−^, the restriction of thermal activation on O_2_^−^ adsorption can be eliminated. Moreover, by loading noble metals Pt and Au on the surface of the oxide, the adsorption barrier can be reduced through the surface catalytic process, and the adsorption of O_2_^−^ can be promoted, leading to RT gas sensing performance.^[^ ^7^, ^11^ ^]^ In addition, the adsorption of O_2_^−^ can also be activated with the local self‐heating effect by designing spatially separated heterojunctions/homojunctions, thereby realizing RT gas sensing.^[^ ^12^, ^13^ ^]^ Although the self‐heating gas sensor still need Joule heating to generate a local high temperature, the heating unit is omitted, which greatly reduces the power consumption of the sensor. Actually, the second strategy does not depend on the unique materials or structures, which has broader application prospects in theory compared with the first one. At present, the reported references demonstrate that the light activation is the only external stimuli strategy instead of the thermal activation to improve the adsorption rate of O_2_^−^.^[^ ^14^ ^]^ For example, the electron‐hole pairs can be generated in wide bandgap semiconductors (ZnO and SnO_2_) by the UV light irradiation, and the induced electrons rapidly reach the surface to form O_2_^−^. Although this method can realize RT gas sensing, it generally requires an external LED light source, which obeys the energy‐saving gas sensors in principle. Therefore, it is still very necessary to develop a novel energy‐saving and high‐speed RT sensing technology.

In 2012, Zhong Lin Wang's group proposed a new triboelectric nanogenerator (TENG) technology based on triboelectrification and electrostatic induction effects.^[^ ^15^ ^]^ In recent years, TENG have been rapidly developed and widely used in low‐frequency mechanical energy harvesting, self‐powered sensing, and other fields.^[^ ^16^ ^]^ In 2018, our group first reported the ionization of air at RT utilizing the voltage as high as several thousand volts of TENG, and the ionization properties and generation mechanism for different gas molecules were also studied.^[^ ^17^, ^18^ ^]^ In 2019, we combined triboelectric plasma with ZnO semiconductor nanodevices to establish a gas‐ionic‐gated (GIG) technology,^[^ ^19^ ^]^ which uses O_2_^−^ gas ions as a floating gate on the semiconductor surface to tune its surface state and electrical transport properties. Based on GIG, the sensitivity of ZnO UV detector can up to 1.3×10^4^ A/W, and the recovery speed can be increased by 946 times. In the ambient environment, the abundant active negative ion clusters (NICs) (OH^−^(H_2_O)_n_, O^−^(H_2_O)_n_, O_2_^−^(H_2_O)_n_, CO_2_^−^(H_2_O)_n_, etc.)^[^ ^20^ ^]^ can finish the modification of ZnO nanomaterials surface state, which provides the possibility for further realization of fast RT gas sensing.

Here, we present a fast RT ZnO‐based VOCs sensor based on self‐powered triboelectric plasma in‐situ regulation technology for the first time. NICs can be generated by using a sliding‐freestanding‐layer TENG and a tungsten needle in air environment with 42% relative humidity (RH), which adsorbs at the homojunctions of ZnO nanowires (NWs) to active the O_2_^−^ ions instead of thermal activation. Based on this, we first obtain a ZnO‐based resistive switch with on‐off ratio as high as 10^5^. Second, the activated ZnO nanodevice shows a sensitivity of 38.3% to 10 ppm acetone gas at RT, and the maximum power consumption is 4.5 mW, while the detection limit is lower than 1 ppm. At the same time, the gas sensor exhibits excellent selectivity to acetone due to the strong interaction between the (0001) polar facets of ZnO and the high electric dipole moment of the acetone. More importantly, the response and recovery times of this sensor are as low as 11 s and 25 s, respectively, which are faster than most of previous reported RT VOCs sensors. We also demonstrate that the adsorbed OH^−^(H_2_O)_4_ at homojunctions of ZnO NWs would undergo the electron transfer at a large bias voltage and form a hydroxyl‐like intermediate state (OH*) on the top of Zn^2+^, leading to the bent of band and increase in conductance, and thus resistive switch. While the RT gas sensing is the result of the combined action of OH^−^ and O_2_ at the homojunctions. The change of the band bend will further affect the ionization state of V_o_ close to OH*, making it easier for the neutral V_o_ to form more active [V_o_^+^−O_2_^−^], thereby achieving RT acetone sensing. This work proposes a novel universal strategy to realize fast RT gas sensing based on active‐ion‐gate.

## 2 RESULTS AND DISCUSSION

**FIGURE 2:**
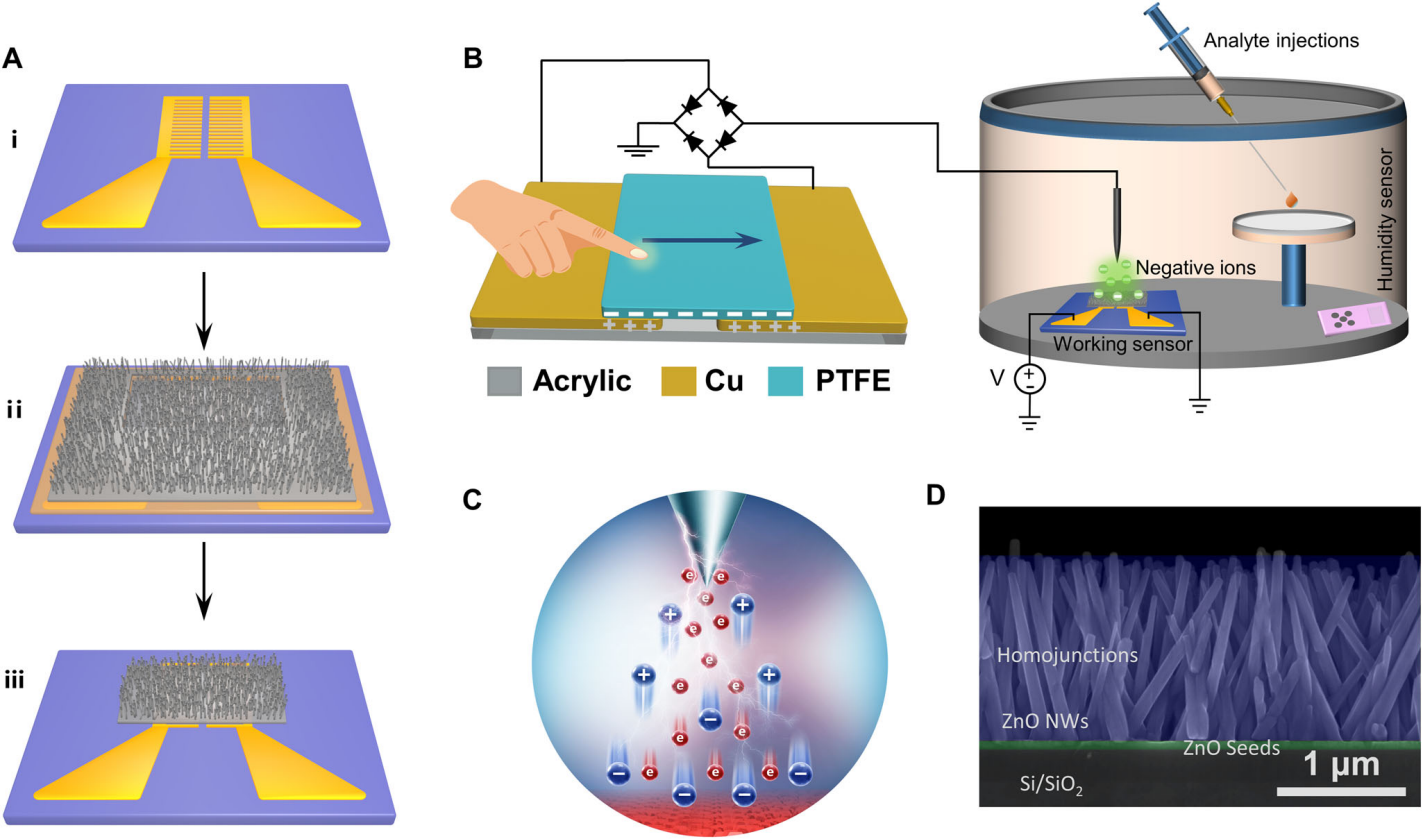
Schematics of the self‐powered triboelectric plasma system. A) Schematic illustrations of ZnO NW devices. B) Three‐dimensional schematic of the triboelectric plasma modulation system that consists of a TENG component, testing cavity, and ZnO NWs devices. C) Schematic diagram of negative ion migration. D) The scanning electron microscopy images of ZnO NWs cross‐section

In this work, the hydrothermally synthesized ZnO NWs without external resistance heater were selected as the modulation and gas‐sensing units. Simplified fabrication process of the ZnO NWs device is shown in Figure 2A. First, a pair of interpolated Au electrodes were fabricated on SiO_2_ (280 nm)/Si substrates by maskless lithography and electron beam evaporation. Second, ZnO seed layer of 300 × 160 μm^2^ was grown on the interpolated Au electrodes by magnetron sputtering, and then ZnO NW arrays were obtained by hydrothermal growth method. Finally, the micro‐area ZnO NW device was obtained by degumming process. We utilized a home‐made triboelectric plasma system to complete the surface states modification of ZnO NWs (Figure 2B). This system integrates a triboelectric plasma generator, a ZnO nano‐device and a sealed chamber. Among them, a sliding freestanding layer TENG was used as self‐powered high‐voltage power source. Rectifier bridge was used to achieve the negative high‐voltage output, and a tungsten needle with diameter of 5 μm was used to generate active NICs. As the tribo‐layer polytetrafluoroethylene (PTFE) of the TENG reciprocating motion, a continuous pulsed negative voltage is applied to the tip of tungsten needle to stimuli corona discharge. The generated NICs would adsorb on the surface of the ZnO NWs under the electric field (Figure 2C), realizing the regulation of the surface states of the ZnO NWs. Furthermore, the camber was used to achieve gas‐sensing detection. Figure 2D shows the cross‐sectional scanning electron microscopy (SEM) image of the gas sensing unit with a vertical laminated structure of Si/SiO_2_/ZnO seeds/ZnO NWs. It is seen that the thickness of the ZnO seeds layer is around 50 nm, and the height of the ZnO NW array is about 1.5 μm. In addition, a large number of ZnO NW homojunctions can be observed, and the numerous gaps between the NWs leave sufficient channels for the adsorption of NICs and gas molecules at the homojunctions (Figure S1, Supporting Information).

The output characteristics of TENG with different external load resistances are shown in Figure S2A, Supporting Information. The open‐circuit voltage and short‐circuit current are 2.5 kV and 2.4 μA, respectively, while the transferred charge is 410 nC per cycle (Figure S2B, Supporting Information). It is easy to achieve corona discharge in air condition by utilizing such stable high‐voltage and low‐current properties of TENG. Furthermore, an externally connected ammeter is to record the generated current by adsorbing NICs at different needle‐plate distances (*d*) per unit time, which is also used to roughly estimate the number of generated NICs. The modulation of ZnO NWs was carried out in air environment with 42% RH. When *d* < 1 mm, a violent spark discharge appears between needle and sample, which can seriously damage the device, similar to previous reports.^[^ ^21^ ^]^ When *d* ≥ 1.25 mm, the induced NICs migrate the surface of ZnO NWs by mild corona discharge, which does not damage the device. As *d* increases from 1.25 to 2 mm, the peak current of corona discharge decreases from 15 μA to 3 μA, and all discharge frequencies are 2 Hz (Figure S2C–E, Supporting Information). The peak current can indirectly reflect the number of NICs induced by TENG, and the amount of NICs decreases with the increase of *d* due to dissipation of electrons with more collision. Therefore, the corona discharge will generate the most NICs per cycle at *d* = 1.25 mm.

**FIGURE 3:**
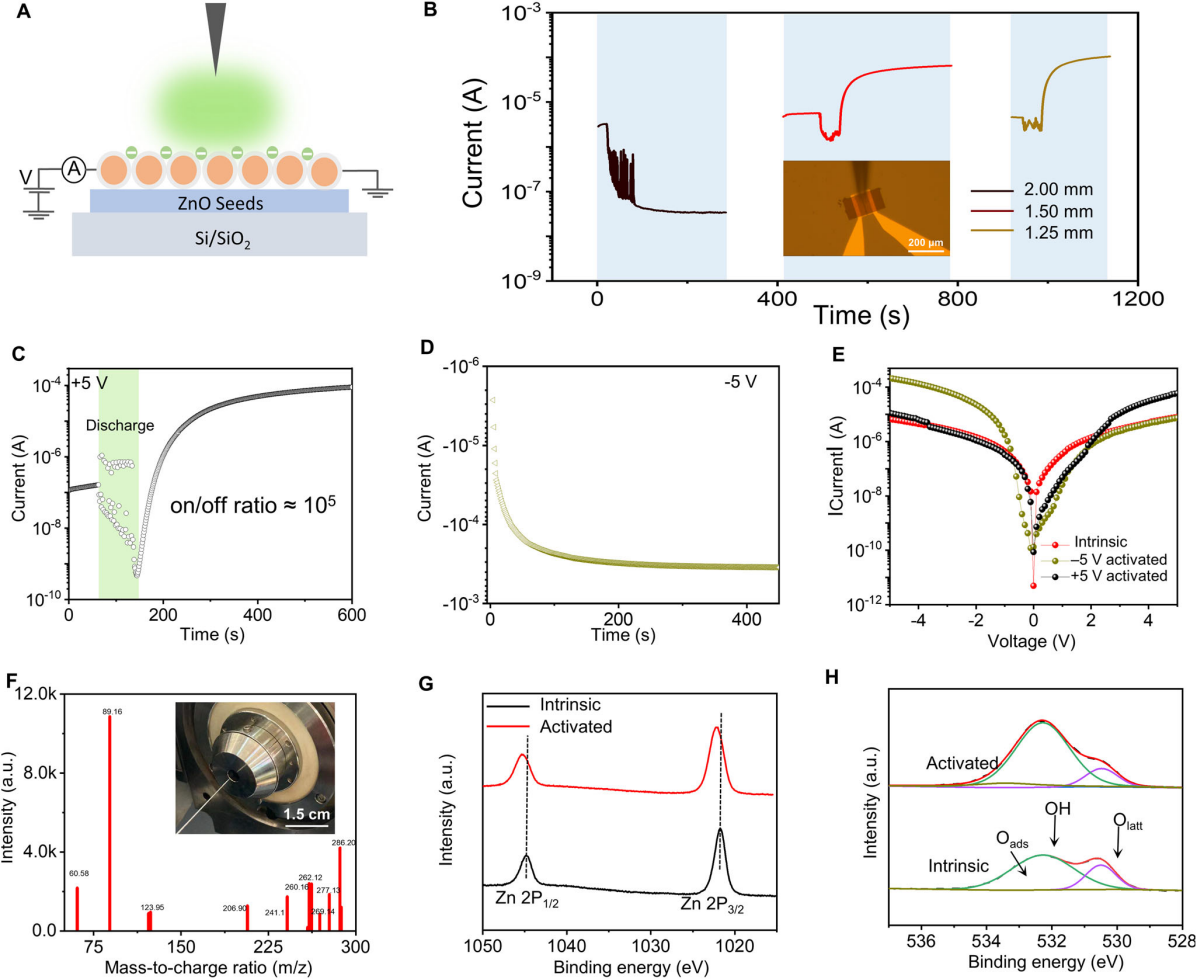
Characteristics of electronics transport, NICs, and ZnO NWs. A) Schematic of the ZnO NWs surface state modulation based on NICs powered by TENG. B) The current response of ZnO NWs after 45 s NICs modulation at various discharge distances (1.25, 1.50, and 2.00 mm). The source‐drain voltage is +5 V. Inset shows a photograph of the in‐situ modulation. C) The current response of the ZnO NWs after 45 s NICs modulation at *d* = 1.25 mm. The source‐drain voltage is +5 V. The on/off ratio is as high as 10^5^ after the NICs modulation. D) The current response of the modulated ZnO NWs as the voltage was quickly switched from +5 V to −5 V. E) The dependence of voltage polarity on the orientation of rectification during negative corona discharge. F) Identification of the NICs species generated at the TENG‐actuated tungsten needle in ambient environment using mass spectrometry. Inset shows a photograph of the test method. G) Zn 2p XPS spectra of ZnO NWs before and after NICs modulation. H) O 1s XPS spectra of ZnO NWs before and after modulation by triboelectric plasma

An abundance of NICs generated by corona discharge in the ambient environment, which can be quickly adsorbed on the ZnO NW homojunctions to regulate their surface state, thereby affecting the electrical transport properties (Figure 3A). Suitable discharge time and *d* are the keys to tune the surface state of ZnO NWs due to the various numbers of adsorbed NICs. Based on the maximum numerous active negative ions of single‐cycle corona discharge at *d* = 1.25 mm, the influence of corona discharge time of 15, 30, and 45 s on the electrical transport properties of the device were investigated, respectively. In order to easily compare, we fixed the sampling time (300 s) after stopping the corona discharge regulation at V_ds_ = 5 V. When a 15 s discharge duration is applied, the current of the device decreases and stabilizes at 2 × 10^−6^ A (Figure S3A, Supporting Information) with the increase of the times, which is consistent with previous GIG phenomenon.^[^ ^22^ ^]^ When a 30 s discharge duration is applied, the current of the device decreases an order of magnitude, and then slightly rises from 3.4 × 10^−6^ A to 4.8 × 10^−6^ A (Figure S3B, Supporting Information) after stopping discharge. As the discharge duration further increases to 45 s, the current first extremely decreases about two orders of magnitude, and then rapidly rises to about 1.5 orders of magnitude above the initial current (Figure S3C, Supporting Information) after stopping discharge, indicating a distinctively resistive switch phenomenon. This is unprecedented that the on‐off ratio greater than 10^3^ by active negative ions adsorption, which is distinctively different from the previous reports.^[^ ^19^ ^]^ The influence of *d* on the electrical performance of the device was further carried out at a discharge duration of 45 s (Figure 3B). When *d* = 2 mm, the current of the device decreases by about 2 orders of magnitude after discharge regulation, and then stabilizes at 3 × 10^−8^ A, serving as GIG. When *d* = 1.25 mm and 1.5 mm, the current of the device decreases first, then increases, and finally stabilizes at about 2 orders of magnitude (10^−4^ A) above the original current, implying an obvious resistance switching phenomenon. Furthermore, Figure S4, Supporting Information shows the resistive switching behavior regulated by 3 discharge cycles, which indicates that the resistive switching behavior is highly repeatable and the discharge regulation is mild. Above all, the obvious resistance switching phenomenon only occurs when a sufficient amount of NICs are adsorbed on ZnO NWs surface during the process of triboelectric plasma modulation.

We perform the discharge regulation on ZnO NWs device at *d* = 1.25 mm again (Figure 3C). It is clearly seen that the on/off ratio is as high as 10^5^ due to the low initial current of ZnO NWs device. After the current rises and stabilizes at 10^−4^ A at +5 V bias voltage, the immediately measured current‐voltage (IV) curve shows rectification phenomenon, and the positive current is higher than the negative current (the rectification ratio is 6.65, Figure 3E). At this time, in order to interpret the electrical transport model and analyze the dependence of the electron transfer at different locations on polarity and voltage, the bias voltage is quickly switched to −5 V. After the current of the device rises and stabilizes at −10^−3^ A (Figure 3D), the immediately measured IV curve also shows the rectification phenomenon, and the negative current is higher than the positive current (the rectification ratio is 17.41, Figure 3E). Clearly, it can be seen that the orientation of rectification relies on the voltage polarity during the corona discharge. When the positive voltage regulation is applied during the corona discharge, the positive current increases, while the negative current does not change significantly. While the negative voltage regulation is applied during the corona discharge, an opposite variation trend appears (Figure 3E). More interestingly, the IV curves before and after discharge regulation at +5 V bias voltage have an intersection at around +2 V. Under the positive bias voltage, the current after discharge regulation is less than the initial state below +2 V, implying that the adsorbed negatively active ions on the ZnO surface are served as GIG. As the bias voltage gradually increases to over +2 V, the current after discharge regulation begins to be higher than the initial state, which indicates the transition from GIG to resistive switch caused by changing surface state of various voltage. A similar phenomenon occurs under the negative bias voltage. This polarity and voltage dependence phenomenon probably due to the back‐to‐back Schottky barrier changes at the homojunctions.

Apart from the amount of NICs, the NICs species also play a crucial role in the modulation of the surface states of ZnO NWs during the regulating process by triboelectric plasma. The actual air composition is complex. In addition to the main N_2_ and O_2_, it also contains a large amount of water molecules and CO_2_. In order to clarify the influences of NICs species on the resistive switch, the gas atmosphere of the discharge environment must be qualitatively investigated. Therefore, we perform the discharge regulation (45 s) tests in pure O_2_, pure N_2_, synthetic air (O_2_ and CO_2_), vacuum and air environment at *d* = 1.25 mm, 1.5 mm, and 2.0 mm, respectively (Figure S5, Supporting Information). It is well known that the discharge model belongs to field emission in vacuum environment. When *d* = 2 mm, a huge amount of electrons emitted by the cold cathode will instantly pierce ZnO NWs, leading to damage of the device and thus the current drops sharply to 0. Since N_2_ are the non‐negatively charged gas molecules, they cannot absorb electrons to form the negative ions, so the discharge mainly produces electrons in highly pure N_2_ environment. However, the numbers of electrons that finally reach the surface of ZnO NWs are limited due to the existence of N_2_. Therefore, the decrease of current is small (about 2 times), which stabilizes at 2×10^−6^ A after the discharge regulation. Accordingly, the influence of electrons on resistive switch can be excluded through the discharge regulation in N_2_ or vacuum conditions. Under pure O_2_ or synthetic air environments, the current has a similar variation trend with the operating of discharge regulation. Therefore, the influence of negative ions, such as the O^−^, O_2_^−^, CO_2_^−^ and CO_3_^−^, on the resistive switch can also be excluded. When *d* = 1.25 mm, the current decreases first and then slowly rises in air environment with RH = 32%. In spite of the fact that the increase is slight, humidity has a certain impact on resistive switch. Furthermore, we compared the regulation results in dry synthetic air and 42% RH synthetic air at *d* = 1.25 mm (Figure S6, Supporting Information), revealing that the device shows obvious resistance switch only the regulation in 42% RH synthetic air. All these results clearly indicate that water molecules are a necessary factor for the resistance switch during the discharge regulation process, and sufficiently ionized water molecules are able to trigger a large resistance switch.

In order to clarify how ionized water molecules affect the resistance switch of ZnO NWs, herein we perform an in‐situ measurement of NICs in air at *d* = 1.25 mm with 42% RH by using mass spectrometry. The results show that the generated negative air ions by the corona discharge mainly include water molecules wrapped with OH^−^, O^−^, O_2_^−^, CO_2_^−^, CO_3_^−^, NO_2_^−^, and HCO_3_^−^ (Figure 3F) clusters. The generated NICs were specific counted, as shown in Figure S7, Supporting Information. It can be seen that the intensity of OH^−^(H_2_O)_4_ clusters is as high as 10663, which means that the OH^−^(H_2_O)_4_ clusters are the most in the negative ions generated by air corona discharge. X‐ray photoelectron spectroscopy (XPS) can reflect more details of the surface chemical compositions and element valence states in materials. XPS spectra of ZnO NW devices before and after the discharge regulation were tested. The binding energies at 1045.1 eV and 1021.9 eV corresponds to Zn 2P_1/2_ and Zn 2P_3/2_, respectively. It can be seen that both the two peaks show obvious blue shift after the corona discharge modulation, which is highly related to NICs adsorbed by the discharge regulation (Figure 3G). The species of oxygen‐containing chemical composition before and after discharge regulation can be evaluated by fitting the O1s core levels with three components (Figure 3H), that is, adsorbed oxygen (O_ads_, peak at 530.5 eV), hydroxyl species (OH, peak at 532.3 eV), and lattice oxygen (O_latt_, peak at 533.4 eV). Accordingly, the relative intensity of OH increases from 74.9% of initial ZnO NWs to 78.4% of regulated ZnO NWs, and the relative intensity of O_ads_ increases from 0.1% of initial ZnO NWs to 9.0% of regulated ZnO NWs (Figure S8, Supporting Information), which indicates that the successful surface modulations of ZnO NWs by the triboelectric plasma regulation. Therefore, combining the electrical transport properties with mass spectrum results, it can easily confirm that OH^−^(H_2_O)_4_ clusters play a crucial role in the surface states modification of ZnO NWs. Generally, ZnO with abundant defects has two emission bands at RT: one is the recombination emission between the intrinsic energy bands of ZnO located in the ultraviolet region; the other is the visible emission caused by defects such as V_o_.^[^ ^23^ ^]^ Therefore, we compared the photoluminescence (PL) spectra of ZnO NWs before and after discharge regulation (Figure S9, Supporting Information). The results show that both the two ZnO samples have strong UV luminescence, but the visible luminescence caused by defects were both equally weak. Clearly, the PL spectra before and after the regulation are not much difference, which indicates that the number of defects of the ZnO NWs does not change significantly by the triboelectric plasma regulation. Since the above experimental results have demonstrated that O^−^ and O_2_^−^ do not lead to resistive switching phenomenon, we can confirm that the key factor affecting the resistance switching performance of the device is the adsorbed OH^−^(H_2_O)_4_ clusters.

**FIGURE 4:**
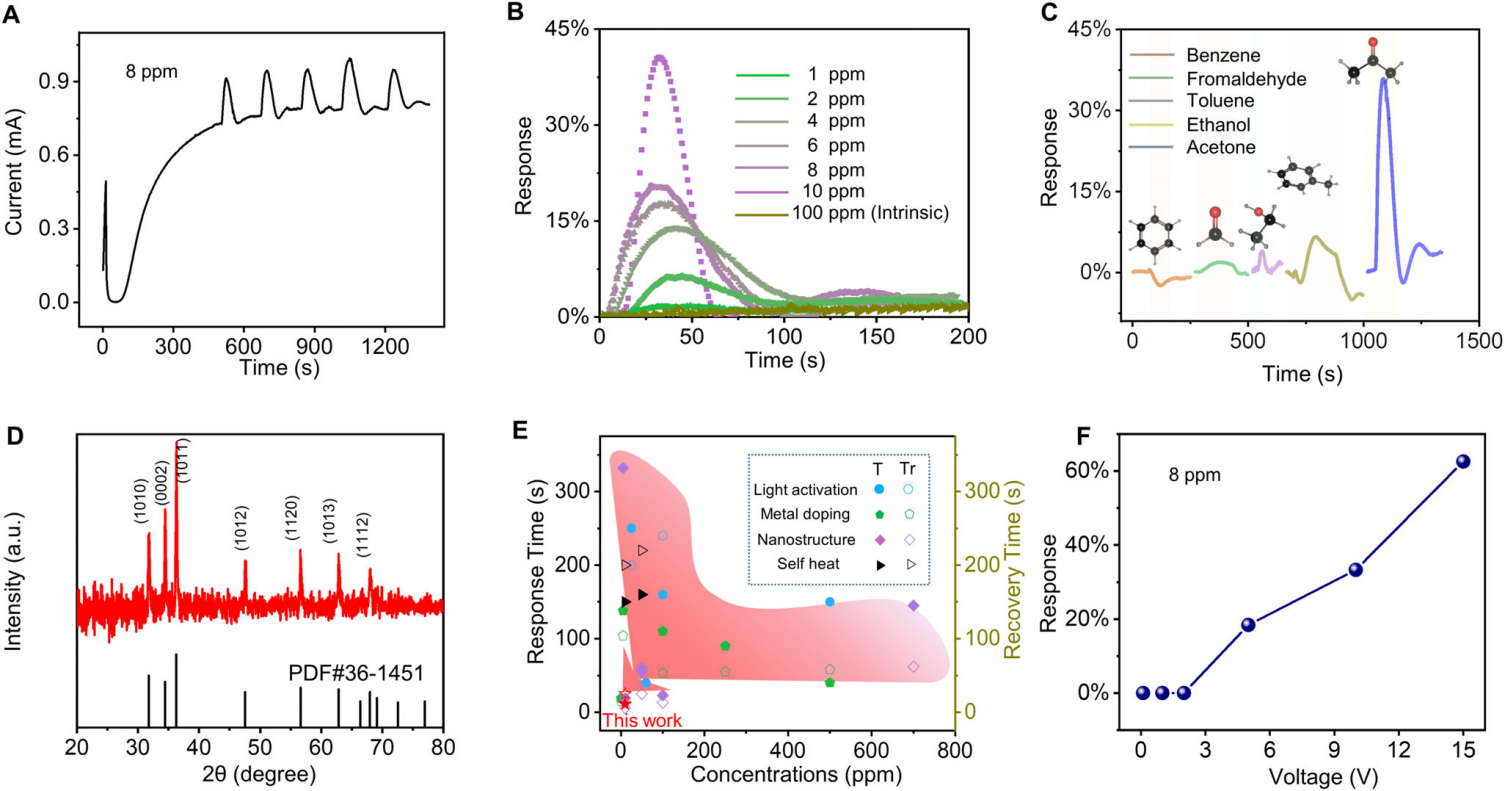
Gas sensing performance of the activated ZnO NWs. A) The response and recovery performance of the ZnO‐based sensor to acetone gas after 45 s NICs modulation at room temperature. B) The sensitivity of the gas sensor to different acetone concentration before and after the NICs modulation. C) The sensitivity of the modulated ZnO sensor to various target VOCs at 10 ppm under room temperature. D) XRD of ZnO NWs and standard ZnO of PDF#36‐1451, respectively. E) Comparisons of the response time (T) and recovery time (Tr) of room temperature VOCs sensors realized by light activation,^[^ ^25^, ^26^, ^27^, ^28^ ^]^ metal doping,^[^ ^29^, ^30^ ^]^ nanostructure,^[^ ^31^, ^32^, ^33^, ^34^ ^]^ and self‐heat effect ^[^ ^12^, ^13^ ^]^. F) Plot of the relationship between 8 ppm acetone response and applied voltage

The RT gas sensing test was carried out in the air environment for generating an obvious resistive switch (*d* = 1.25 mm, RH = 42%, and 45 s discharge time). As the current was gradually stabilized at 0.7 mA, 8 ppm of acetone gas was introduced to conduct the gas response test (Figure 4A). It can be seen that the current of the sensor will rise immediately once the acetone gas is introduced. After opening the chamber cover, the current quickly recovers to 0.7 mA, and the response is stable, which can realize multiple cycles of detection. Therefore, the sensing of acetone gas at RT was successfully realized by the adsorption of active NICs on ZnO NWs. The response of gas sensor to different concentrations acetone gas at RT was measured, as shown in Figure 4B. The initial sensor does not respond to even 100 ppm acetone gas, and the IT curve of the device maintains a horizontal straight line when the gas was introduced, which is in concert with the reported ZnO NWs gas sensor with the same structure.^[^ ^8^ ^]^ However, the regulated ZnO NWs by triboelectric plasma shows excellent sensitivity of 1.7%, 5.4%, 10.3%, 19.8%, 25.3%, and 38.3% to 1 ppm, 2 ppm, 4 ppm, 6 ppm, 8 ppm, and 10 ppm acetone gas, respectively. Figure S10, Supporting Information shows the multi‐cycle test of different concentrations acetone sensing, which means that the regulated ZnO NWs sensor has good stability. Furthermore, the response of activated ZnO NWs to different VOCs analytes under 10 ppm concentrations was measured, as shown in Figure 4C. It is clearly shown that the activated ZnO NWs exhibits excellent selectivity toward acetone as compared to other VOCs. One suitable explanation is that the specific polarity facets (0001) with spontaneous local electric dipole moment in ZnO nanocrystals have strong interactions with the large dipole moment acetone gas,^[^ ^24^ ^]^ thus improving the preferential adsorption capacity of acetone gas on the surface. X‐ray diffraction (XRD) patterns can show the intensities of the individual crystal planes, and the ratio of the (0002) and (1010) peaks intensity can reflect the degree of (0001) polar facets exposure. The results show that the I_0002_/I_1010_ = 1.27, which means that the (0001) crystal plane has a higher degree of exposure than the standard ZnO (Figure 4D). Since the dipole moment of acetone (2.88 D) is larger than that of methanol (1.70 D), formaldehyde (2.33 D), benzene (0 D), ethanol (1.69 D), and toluene (0.38 D), the interaction between ZnO and acetone molecules are stronger than that of other VOCs. However, the experimental phenomena observed for formaldehyde and toluene are slightly inconsistent with the theory, which may be due to the slight errors in the test process, caused by the different saturated vapor pressures of various organic gases. In conclusion, the triboelectric plasma‐tuned gas sensor exhibits high selectivity for acetone. Figure 4E and Table S1, Supporting Information summarizes the response time and recovery time of the currently developed RT ZnO‐based sensor for VOCs sensing.^[^ ^12^, ^13^, ^25^, ^26^, ^27^, ^28^, ^29^, ^30^, ^31^, ^32^, ^33^, ^34^ ^]^ Our triboelectric plasma‐tuned sensor exhibits excellent performance with the fast response (11 s) and recovery (25 s) at the same time (Figure S11, Supporting Information), which are comparable to, if not better than, existing RT VOCs sensors. Such a fast response may be due to the easy adsorption and reaction of active ions on the ZnO surface. It is noteworthy that there is an optimal humidity regulation range, 42–63%. There is no obvious resistive switching and room temperature acetone responses outside this optimal humidity range due to the fact that too few water molecules are used for ionization to generate OH^−^ or too many water molecules form a water film on the surface of ZnO.

A further investigation revealed that the gas sensing is dependent on the applied bias voltage (Figure 4F). When the bias voltage is less than +3 V, the device does not response to acetone gas. As the applied bias voltage is greater than +3 V, the device generally exhibits a response to acetone gas at RT, implying that the large bias voltage may be more favorable for the adsorption of reactive active ions on the ZnO surface. This phenomenon is in concert with the above results in Figure 3E, that is, the resistance switch is in dependent on the larger voltage after the discharge regulation. At this time, the gas‐sensing response gradually increases with increasing the Voltage (the highest sensitivity is 62% at +15 V), which is consistent with reported self‐heating phenomenon.^[^ ^13^ ^]^ Furthermore, a similar phenomenon was observed when a negative voltage is applied. In order to verify whether the gas response at RT of the triboelectric plasma‐tuned sensor is a self‐heating effect, several corresponding controlled experiments were carried out. When the current above the initial value after the discharge regulation, an obvious RT acetone response can be observed. Then immediately heating the device from RT to 60°C, the current increases slightly (the amplitude is about 20%). As a further rise of temperature from 60°C to 120°C, the current drops rapidly and finally stabilizes at 7 × 10^−5^ A. At this time, the device does not respond to 100 ppm acetone even the temperature is as high as 120°C (Figure S12, Supporting Information), and the resistance switch disappears. That is to say, the adsorption of OH^−^(H_2_O)_4_ clusters on ZnO NWs is relatively stable at RT, but the desorption occurs easily at a relative higher temperature, which contradicts the previously reported self‐heating effects that the sensitivity of self‐heated device increases almost linearly with rising the temperature.^[^ ^12^ ^]^ Furthermore, the thermal infrared imaging shows that the temperature of the ZnO NWs before and after the regulation do not change significantly (Figure S13, Supporting Information), which directly confirmed our inference. Accordingly, although the gas sensing performance at RT has similar voltage dependence, the self‐heating effect can be ruled out by designing the control experiments.

We have found that the adsorption of OH^−^(H_2_O)_4_ clusters plays a crucial role in the resistive switch and RT gas‐sensing of ZnO NWs. However, it is well known that adsorbed O_2_^−^ is the key factor affecting gas sensing performance of ZnO NWs. Therefore, we further investigated whether O_2_ molecules also play a key role in RT gas sensing performance of triboelectric plasma activated sensor. Figure S14, Supporting Information summarizes the effects of different discharge and test environments on resistive switch and RT acetone response, respectively. It can be seen that a sufficient amount of water molecules in the discharge environment is necessary for the device to generate resistive switch, but not ensure the generation of RT acetone sensing. Actually, RT acetone sensing can only occur if the discharge is carried out in an environment with sufficient water molecules and detection in an environment containing oxygen, demonstrating that RT acetone response is the result of the combined action of OH^−^ and O_2_ on the ZnO surface. For example, the change trend of the current in pure N_2_ environment with RH = 42% is the same as the result obtained in the air environment with the same humidity, and then the response to acetone gas appears with gradually introducing O_2_ (Figure S15, Supporting Information). This means that the adsorption of OH^−^ can indirectly promote the adsorption of O_2_ molecules on the ZnO NWs, thus leads to the obvious redox reaction with acetone, further changing the conductance of ZnO materials and realizing RT gas sensing. This result is consistent with the front of XPS that the amount of OH and O_ads_ increases simultaneously after triboelectric plasma regulation. It is noteworthy that the increasing of O_ads_ not only originates activated ones with OH^−^, but also comes from O^−^ and O_2_^−^ generated by corona discharge.

**FIGURE 5:**
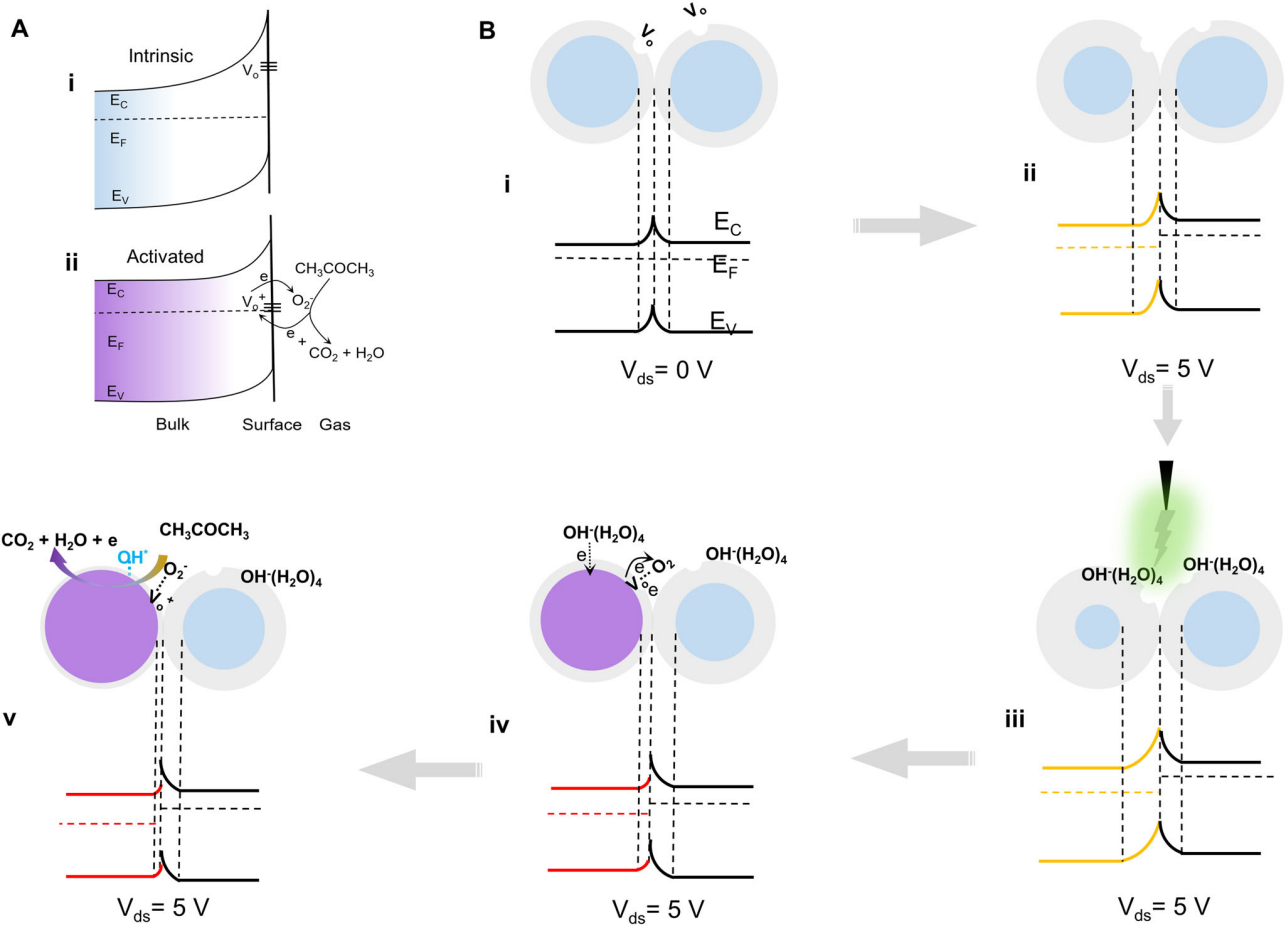
Analysis of the physical mechanism of back‐to‐back ZnO NWs in the activation process by using self‐powered triboelectric plasma. A) The surface band diagram of ZnO NW before and after the activation of triboelectric plasma. B) Schematic and band diagram of barrier models of simplified back‐to‐back ZnO NW homojunctions before and after the modulation of triboelectric plasma

Generally, there are two kinds of O_2_^−^ ions on the surface of ZnO at RT: one is that O_2_ directly gets electrons from the conduction band to form [C−O_2_^−^], which adsorbs on the surface of ZnO; the other is that O_2_ takes away electrons from V_o_ to form [V_o_^+^−O_2_^−^]. The previous reports demonstrate that spontaneously adsorbed O_2_^−^ on the vacancies has higher activity than [C−O_2_^−^].^[^ ^35^, ^36^ ^]^ For photodetector, [C−O_2_^−^] plays a dominant role in the photodetection process because the number of [C−O_2_^−^] is much larger than that of [V_o_^+^−O_2_^−^].^[^ ^18^ ^]^ However, for gas sensing or photocatalysis, the [V_o_^+^−O_2_^−^] is much easier to stabilize and react with the reductive gas due to the lower energy barrier in the reaction kinetics compared with [C−O_2_^−^],^[^ ^37^ ^]^ resulting in dominance. This theory is in concert with our experimental phenomenon. Besides the OH^−^ clusters, there are also active O_2_^−^ and O^−^ clusters adsorbed on the surface of ZnO. However, these non‐spontaneous active ions [C−O_2_^−^ or V_o_−O_2_^−^] do not lead to resistive switch and RT gas sensing. The above experimental results show that the emergence of RT gas sensing is the result of the combined action of OH^−^ and O_2_ on the ZnO NWs, and the adsorption of OH^−^ can indirectly promote the spontaneous adsorption of O_2_ on the ZnO NWs. In fact, the adsorbed OH^−^ will undergo electron transfer at a large bias voltage, and forms a hydroxyl‐like intermediate state (OH*) on the top of Zn^2+^, leading to the bent of band and increase in conductance.^[^ ^38^ ^]^ The change of the band bend will further reduce the potential barrier required for V_o_ ionization close to OH*, making it easier for the electrically neutral V_o_ to form more active [V_o_^+^−O_2_^−^], thereby achieving RT acetone sensing (Figure 5A).

The mechanism of the RT acetone sensing of ZnO NWs by the triboelectric plasma regulation can be explained by using the schematic illustration and energy band diagram in Figure 5B. The cross‐contacted ZnO NW homojunctions is equivalent to a back‐to‐back Schottky barrier. When V_ds_ = 0 V, the potential barriers are the same on both sides of the homojunctions (Figure 5Bi). In the non‐equilibrium state, that is, when V_ds_ = 5 V, the conductance of the entire device is controlled by the reverse‐biased barrier, and the voltage drop is concentrated on the left side, leading to increase of barrier and decrease of Fermi level (Figure 5Bii). As the corona discharge regulation is applied, the adsorbed OH^−^(H_2_O)_4_ at the homojunctions can be equivalent to GIG to simultaneously increase the back‐to‐back Schottky barrier height (Figure 5Biii). Electrons can transfer from OH^−^ to the left ZnO NW after the discharge regulation, presumably due to the Fermi level of the left ZnO is lower than the HOMO level of OH^−^(H_2_O)_4_ (Figure S16A, Supporting Information). The formed OH* will take down the energy band, leading to the increase of conductance of the left ZnO. On the other hand, the Fermi level of right ZnO is higher than the HOMO level of OH^−^(H_2_O)_4_ due to without voltage drop on the right side(Figure S16B, Supporting Information), and electrons cannot be transferred to the ZnO NWs due to the high barrier, only serving as GIG. The adsorbed OH* accelerate the ionization of the neighboring V_o_ to generate V_o_^+^, while the electrons generated by the ionization induce the adsorption of more O_2_ in the air, resulting in sharp increase of [V_o_^+^−O_2_^−^] (Figure 5Biv). The increased [V_o_^+^−O_2_^−^] reacts with acetone at RT, and releases an electron to V_o_^+^, which further increases the conductance of ZnO, thereby realizing RT acetone multi‐cycle sensing (Figure 5Bv).

## 3 CONCLUSION

In summary, we report a novel strategy to achieve rapid RT gas sensing of ZnO NWs by using self‐powered triboelectric plasma system. Our findings highlight the direct impact of OH^−^(H_2_O)_4_ on the ionization of V_o_ neighboring ZnO NWs homojunctions, and provide important guidance for eliminating the restriction of thermal activation on O_2_^−^ adsorption. The adsorbed OH^−^(H_2_O)_4_ accelerate the ionization of the neighboring V_o_ to generate V_o_^+^ by electrons transfer, resulting in boosting of [V_o_^+^−O_2_^−^], thus realizing the RT gas sensing. The activated ZnO NWs shows considerable sensitivity, excellent selectivity, and rapid response to acetone gas at RT with maximum power consumption only 4.5 mW. Our sensor can be well worked in ambient condition due to the unique interaction between OH^−^(H_2_O)_4_ and Zn^2+^, which shows great potential for wide application. The progress of active‐ion‐gate instead of thermal activation has been demonstrated a feasible approach to realize RT gas sensing, which paves the way for boosting the RT gas sensing performance of traditional MOS by customizing specific ions with various discharge gas atmosphere.

## 4 EXPERIMENTAL SECTION

### 4.1 Construction of the self‐powered triboelectric plasma and sensing test platform

Self‐powered triboelectric plasma source was obtained by using a sliding freestanding layer TENG, a rectifier bridge and a discharge tungsten needle. Tribo‐layer was made of PTFE, and electrode layer was composed of two copper foils. The electrical output of TENG was converted into unidirectional negative electrical pulse signal through a full‐wave rectifier bridge. Negative high voltage was then transferred to a tungsten needle to stimuli a mild negative corona discharge by controlling the discharge d, and the *d* was fine‐tuned by a 3D displacement stage. The in‐situ gas sensing test platform was built by a closed cavity, a self‐powered triboelectric plasma source and ZnO NW device.

### 4.2 Fabrication of gas sensor

First, the sliced SiO_2_/Si pieces with a size of 1.5 × 1.5 cm^2^ were placed in acetone, ethanol and deionized water ultrasonically for 5 min, respectively, and then dried with N_2_. Second, the photoresist (AZ 1500, USA) was spun onto the sliced SiO_2_/Si substrate by a spin processor at a fixed rotating speed of 4000 rpm for 60 s, followed by 60 s baking with hot plate at 100°C. The interpolated pattern was obtained by maskless lithography system (DWL 66^+^, Germany), then the substrate was immersed with developer (AZ 400k, USA) for 60 s and dried with N_2_. 5 nm Gr and 65 nm Au were sequentially evaporated on the substrate with an electron beam evaporator (TEMD500, China), and the residual photoresist was removed with degumming fluid to obtain an pair of interpolated Au electrode arrays. Finally, a size of 300 × 160 μm^2^ blank area on interpolated Au electrode arrays was obtained by the photolithography and developing process. ZnO seeds layer with a thickness of 50 nm was deposited by RF magnetron sputtering (the deposition was done at 80 W with 5 mtorr Ar atmosphere for 25 min, PRO Line PVD75, USA). Subsequently, ZnO NWs were grown via the previous reported hydrothermally method. Last, the micro‐area ZnO NW array device was obtained by the lift‐off of residual photoresist in acetone or degumming fluid.

### 4.3 Gas concentration distribution, calculation of sensitivity, and power consumption

Various concentrations of VOCs were prepared by a stationary state gas distribution method. A micro syringe was used to quantitatively obtain different concentrations of VOCs by controlling the injection volume in the sealed chamber. The quantitative gas distribution formula at standard atmospheric pressure is as follows:

(1) $$\begin{array}{c} V_{x}=\left(V\times C\times M\right)/\left(22.4\times D\times P\right)\times \left(273+T_{r}\right)/\left(273+T_{b}\right) \end{array}$$

where *V*_x_ is the target organic liquid volume, V is the volume of test chamber, M is the molecular mass of specific organic molecular, C is the organic gas diffuse concentration in a sealed test chamber, which can be expressed in parts per million concentration (ppm), P is the purity of the organic liquid, D is the density of the organic liquid (g/cm^−3^), and *T_r_* 、*T_b_* are the surrounding ambient temperature and the test temperature inside the chamber, respectively.

The sensitivity can be defined as the relative change of the ZnO NW sensor resistance to the VOCs sensing at RT, which is calculated with the following formula:

(2) $$S=\Delta R/R_{g}=\left(R_{a}-R_{g}\right)/R_{g}$$

where ΔR is the resistance difference of gas sensor in the air and the target organic gas environment; *R_a_* is the resistance of the gas sensor in the ambient environment; *R_g_* is the stabilized resistance of gas sensor in the target organic gas.

The maximum power consumption to 10 ppm acetone gas was estimated as P_max_ = UI, where U = 5 V is the applied drain voltage, I = 0.9 mA is the response current to 10 ppm acetone through the device.

### 4.4 Characterization

The surface and cross‐section morphology of ZnO NWs were examined by the optical microscope (Motic PSM‐1000, China) and SEM (JSM‐7900F, Japan), respectively. The NAIs were quantified and identified by mass spectrometer (AmaZon SL, Germany).The crystalline intensity was measured by XRD (Bruker D8 VENTURE, Germany). The chemical binding states were analyzed by XPS (AXIS ULTRA, English). The output current/voltage, transferred charge and discharge current of the TENG were recorded by the electrometer (Keithley 6514, USA). The electrical performances of the ZnO NWs were measured by the multifunctional semiconductor Tester (Keithley 4200‐SCS, USA).

## CONFLICT OF INTEREST

The authors declare no conflict of interest.

## Supporting information

Supporting Information

## Data Availability

All data of this work are present in the article and the Supporting Information. The other data that support the findings of this work are available from the corresponding author upon reasonable request.
