# Supplementary material for: Active‐ion‐gated room temperature acetone gas sensing of ZnO nanowires array
Source: Exploration (Beijing). 2022 Oct 14;2(6):20220065. doi: 10.1002/EXP.20220065 (PMC10191029; doi:10.1002/EXP.20220065)
Supplement: Supplementary file 1 — Supporting Information [file EXP2-2-20220065-s001.doc]

**Supplementary Information**

**Active-ion-gated room temperature acetone gas sensing of ZnO nanowires array**

Junmeng Guo^1^, Jiahui Gan^1^, Haoran Ruan, Xiaobo Yuan, Chuiyun Kong, Yang Liu, Meiying Su, Yabing Liu, Wei Liu, Bao Zhang, Yongle Zhang, Gang Cheng*, Zuliang Du*

Key Lab for Special Functional Materials, Ministry of Education, National & Local Joint Engineering Research Center for High-efficiency Display and Lighting Technology, School of Materials Science and Engineering, and Collaborative Innovation Center of Nano Functional Materials and Applications, Henan University, Kaifeng 475004, China

Email: [chenggang@henu.edu.cn](mailto:chenggang@henu.edu.cn); [zld@henu.edu.cn](mailto:zld@henu.edu.cn)

^1^Junmeng Guo and Jiahui Gan contributed equally to this work.

**FIGURES AND TABLES**

**
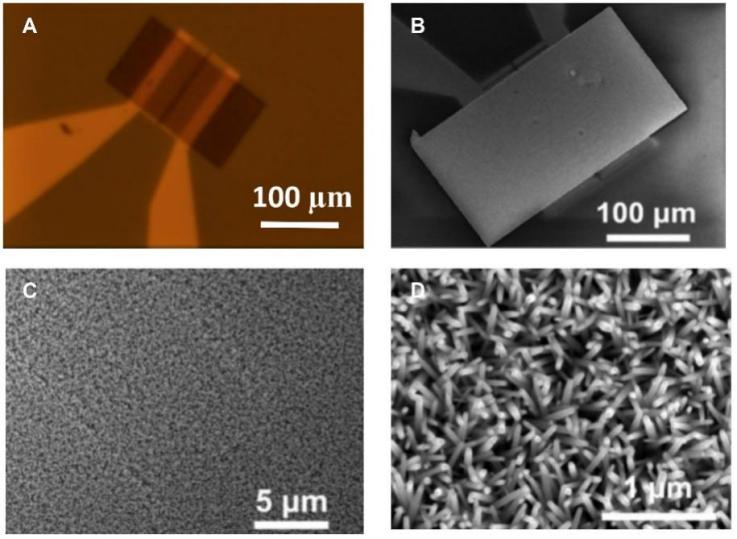
**

**FIGURE S1** (A) Optical photo of ZnO NW device. (B) SEM image of ZnO NW device. (C, D) SEM images of ZnO NW.


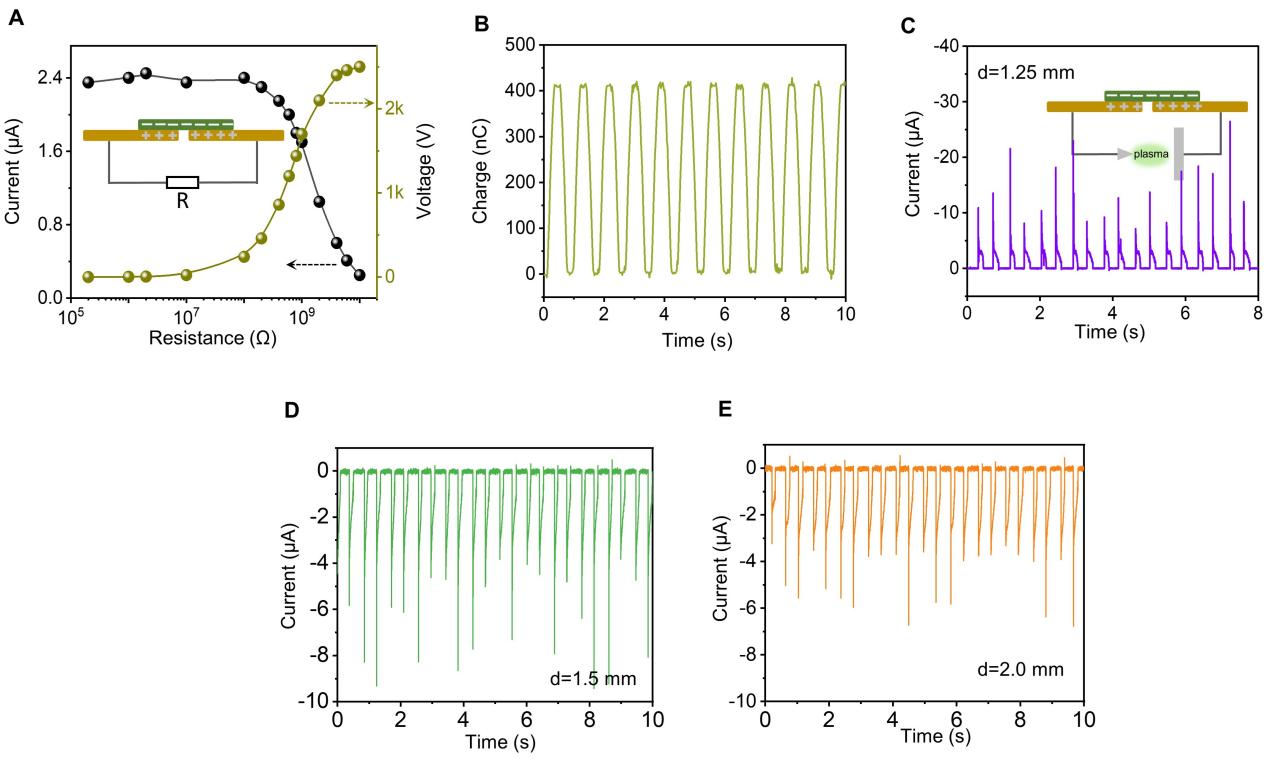


**FIGURE S2** (A) Output current and voltage of the TENG at different load resistances. Inset shows the test schematic. (B) Transfer charge of the TENG. (C) Output current curves of negative corona discharge powered by TENG at d = 1.25 mm . Inset shows the test schematic. (D, E) Output current curves of negative corona discharge powered by TENG at d = 1.5 and 2.0 mm, respectively.


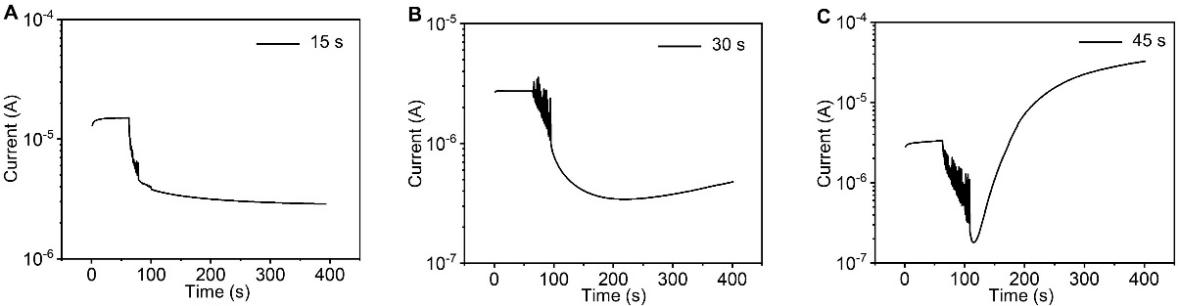


**FIGURE S3** The influence of discharge times on electrical performance of ZnO NWs at d = 1.25 mm.


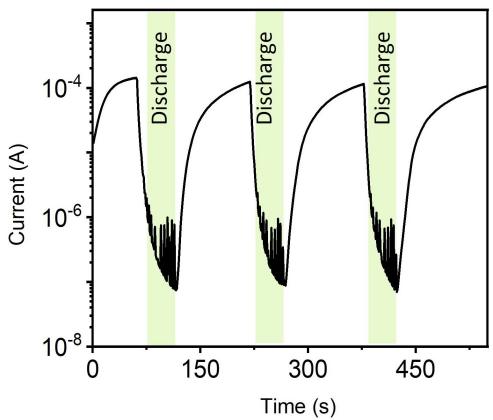


**FIGURE S4** Stability test during the modulation of ZnO nanwires at d = 1.25 mm.


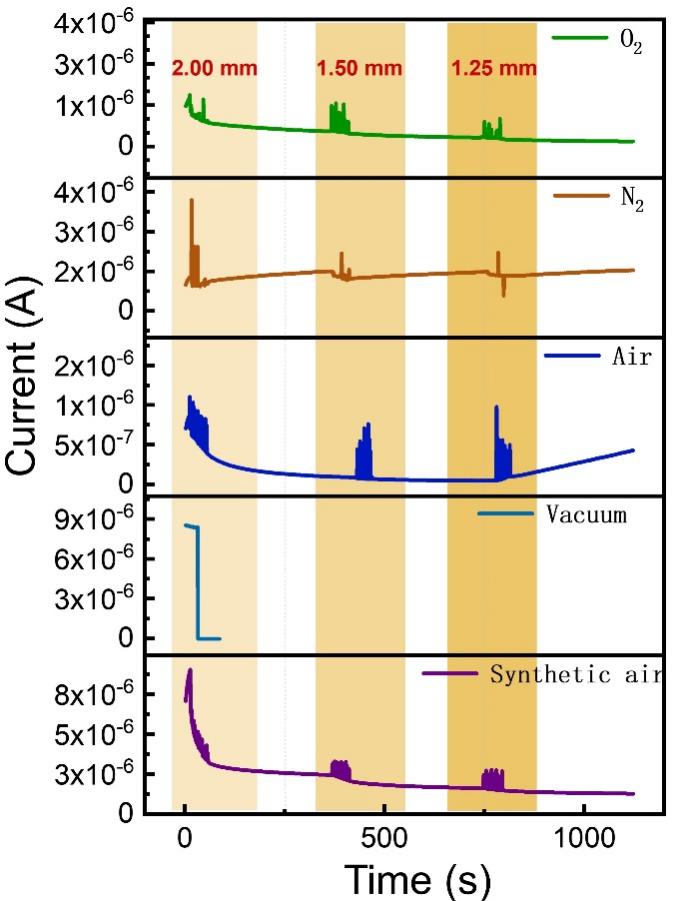


**FIGURE S5** Influence of gas atmosphere and discharge distance on the electrical properties of ZnO NWs.


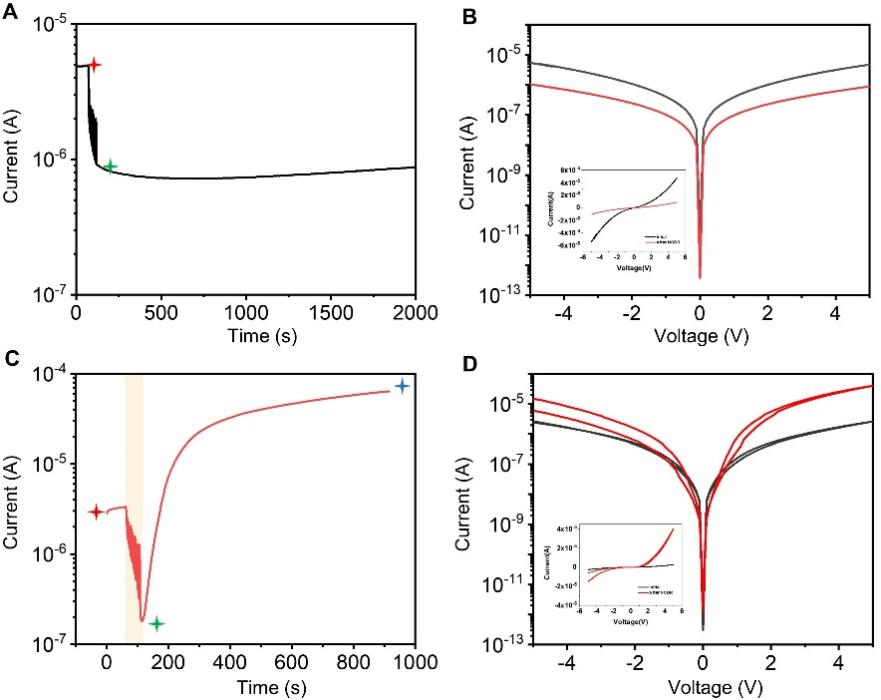


**FIGURE S6** Influence of humidity on the modulation of ZnO NW Devices. (A) The current–time of ZnO NWs with negative corona discharge modulation (d = 1.25 mm, 45 s) under dry air environment. (B) The I-V curves of ZnO NWs before and after discharge modulation of A. (C) The current–time of ZnO NWs with negative corona discharge modulation (d = 1.25 mm, 45 s) under real air environment with RH = 42%. (D) The I-V curves of ZnO NWs before and after discharge modulation of C.


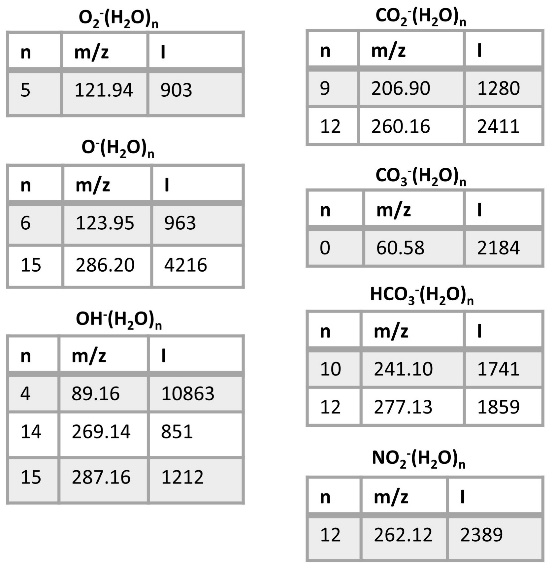


**FIGURE S7** Intensity of TENG generated NICs by mass spectrometry.


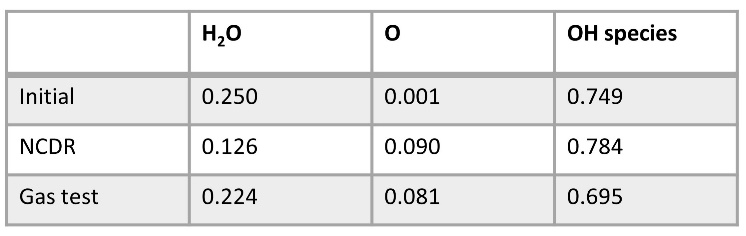


**FIGURE S8** XPS data for ZnO NW before and after negative corona discharge modulation under real environment with an operating voltage of 5 V.


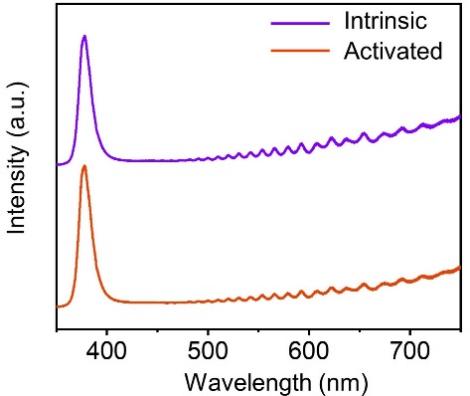


**FIGURE S9** PL spectra of ZnO NWs before and after modulation by triboelectric plasma.


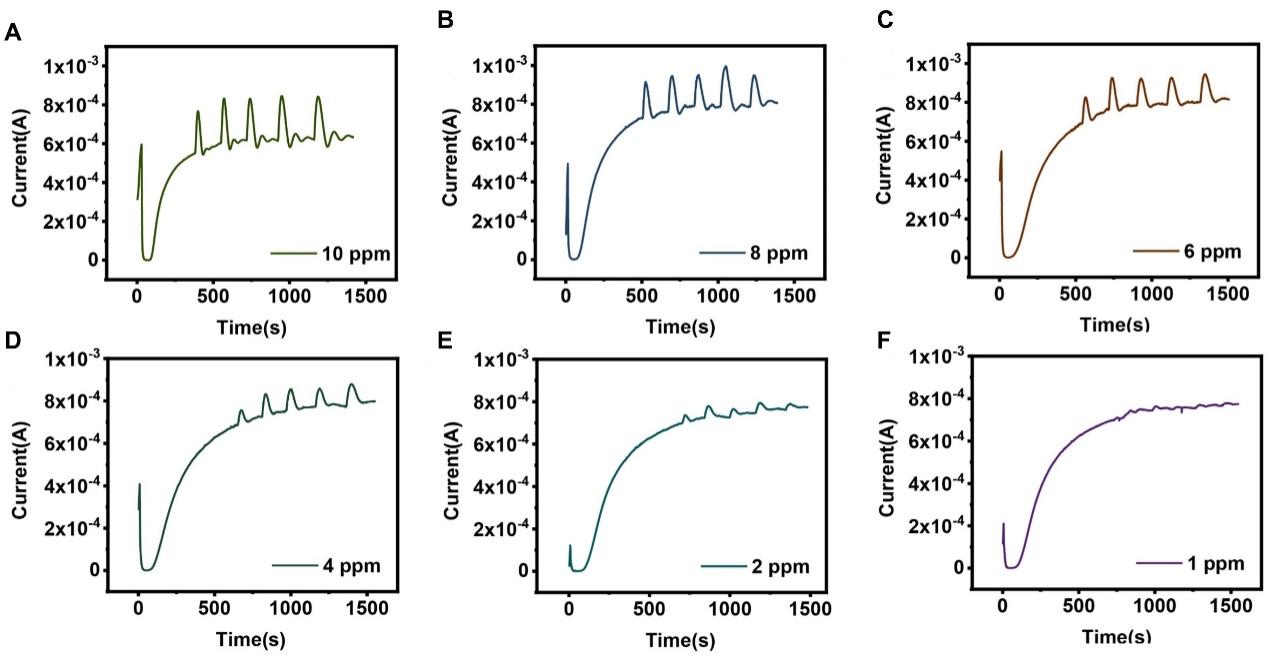


**FIGURE S10** Room-temperature response of ZnO NW sensor under various acetone concentration (1, 2, 4, 6, 8, 10 ppm, respectively.) after negative corona discharge modulation.


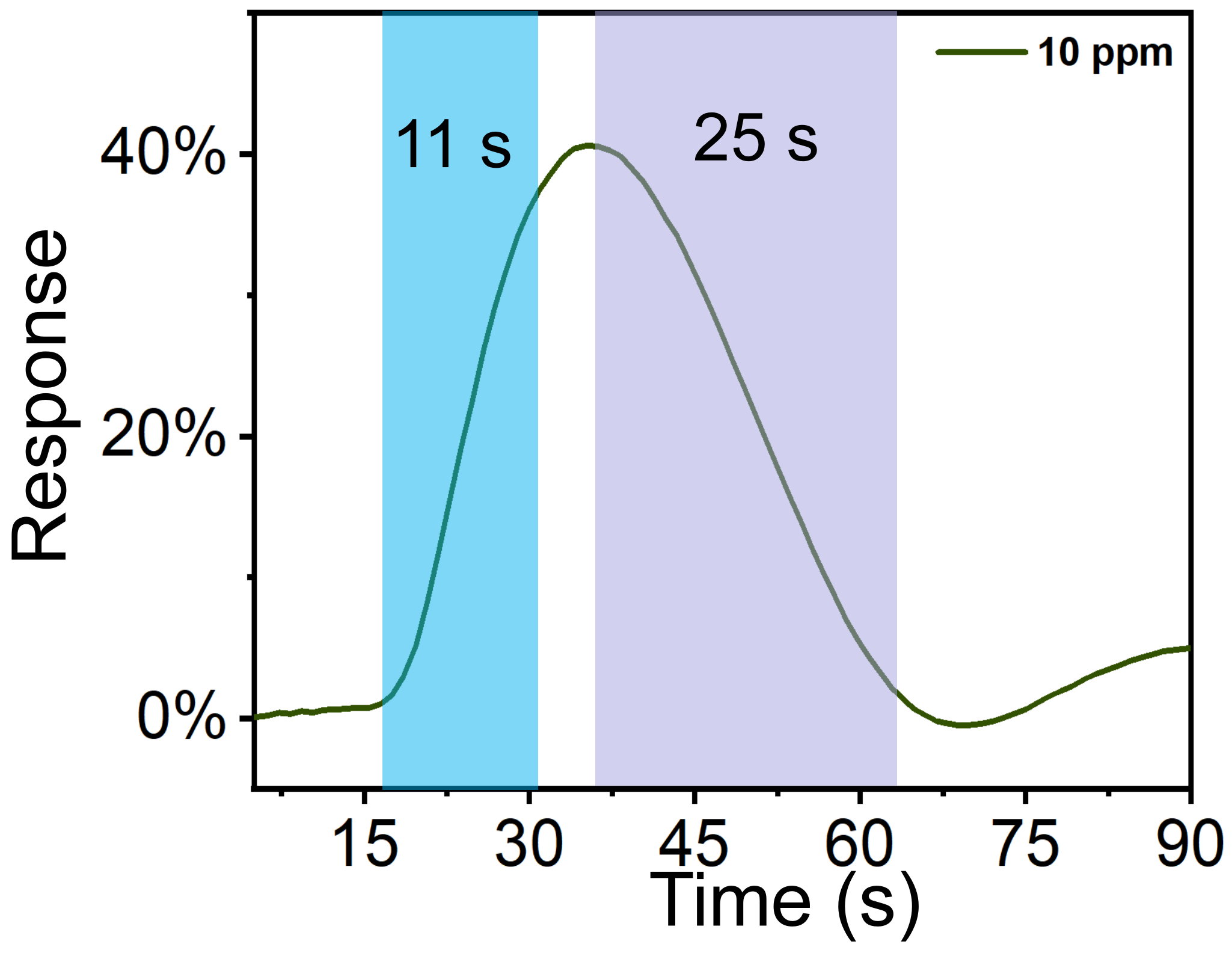


**FIGURE S11** Response and recovery time of negative ion activated ZnO NW device to 10 ppm acetone at room temperature.


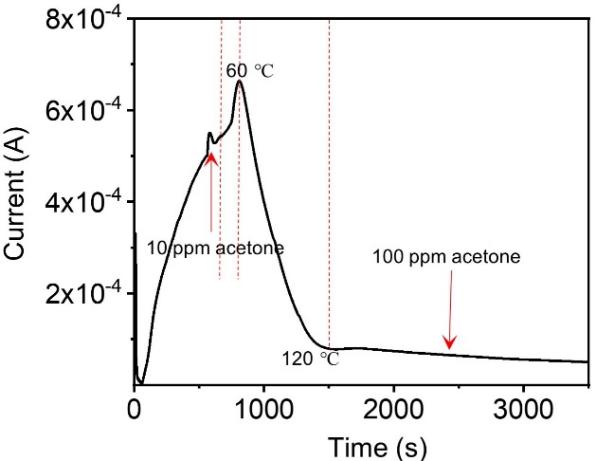


**FIGURE S12** The response of ZnO NWs with negative corona discharge modulation under real air environment first, and then gradually increase temperature to 120 ℃.


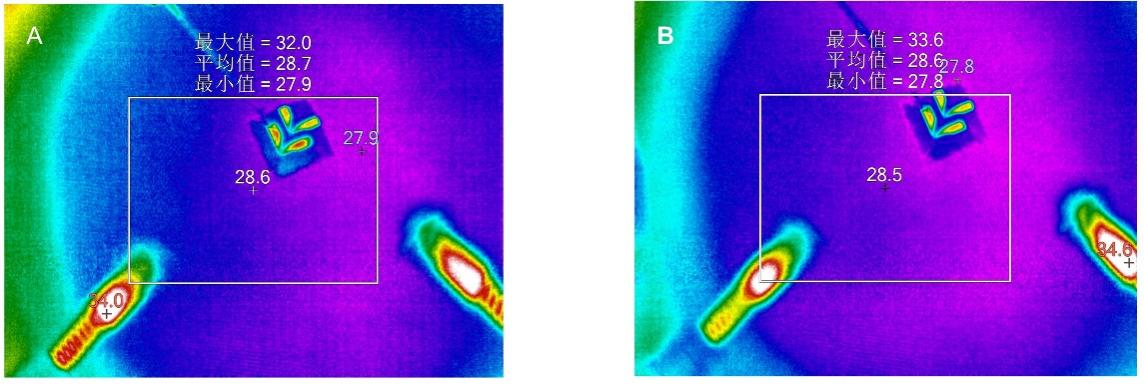


**FIGURE S13** Thermographic images of ZnO NW sensors before (A) and after (B) negative corona discharge modulation under real environment with a operating voltage of 5 V.


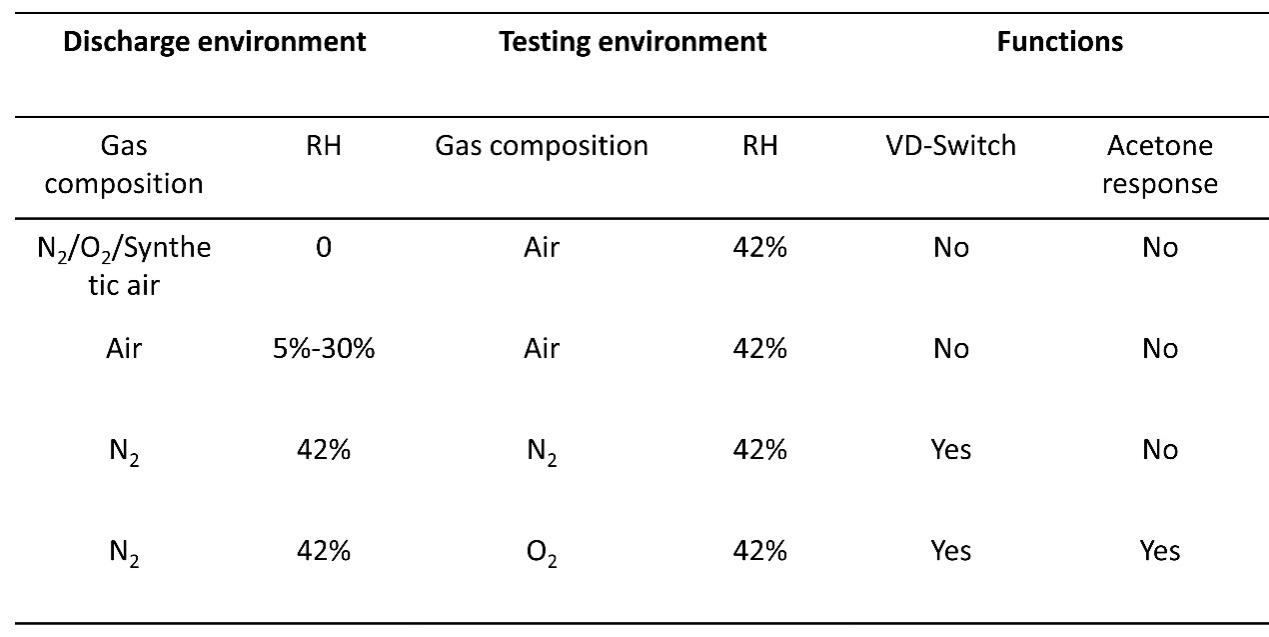


**FIGURE S14** The influence of discharge and test environments on resistive switch and acetone response.


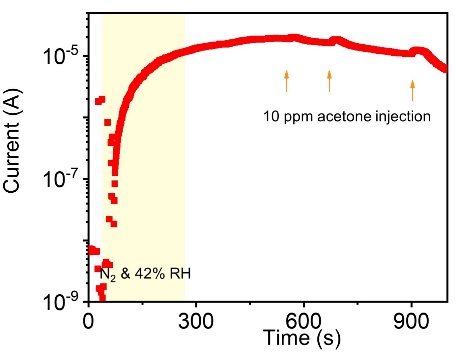


**FIGURE S15** The current–time of ZnO NWs with negative corona discharge modulation under N_2_ & 50% RH environment, and then gradually increase O_2_ to the cavity to test RT gas response.


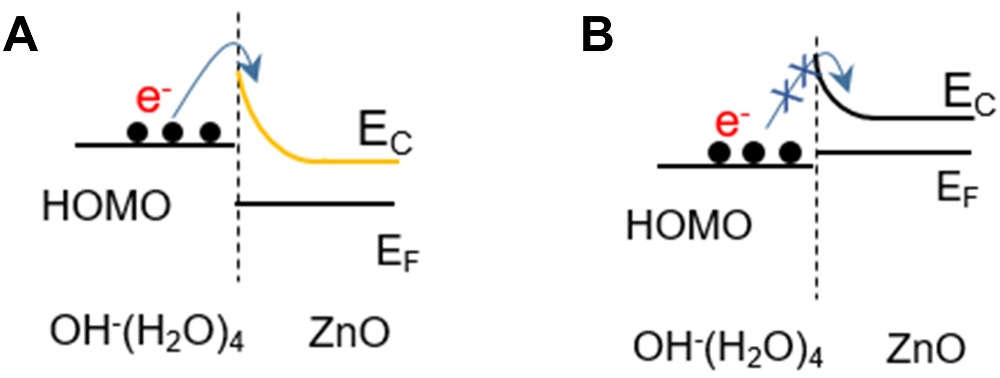


**FIGURE S16** The band diagram of the electrons transfer path from OH^−^(H_2_O)_4_ to ZnO NWs.

**TABLE S1 The summary of the response and recovery time of the currently developed RT ZnO-based sensor for VOCs sensing.**

**
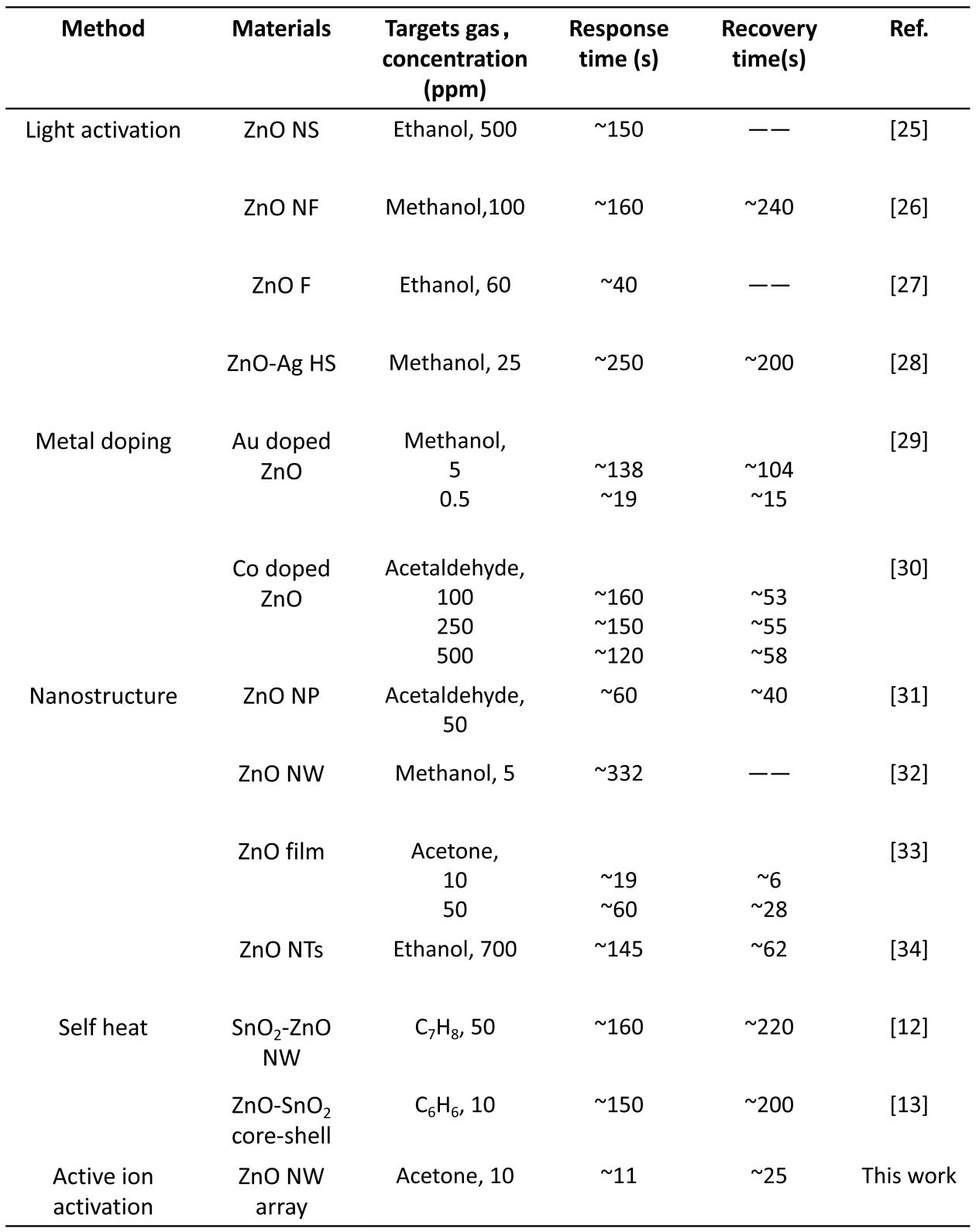
**
